# Macrophages reprogramming driven by cancer-associated fibroblasts under FOLFIRINOX treatment correlates with shorter survival in pancreatic cancer

**DOI:** 10.1186/s12964-023-01388-7

**Published:** 2024-01-02

**Authors:** Zainab Hussain, Thomas Bertran, Pascal Finetti, Eugenie Lohmann, Emilie Mamessier, Ghislain Bidaut, François Bertucci, Moacyr Rego, Richard Tomasini

**Affiliations:** 1 Cancer Research Center of Marseille, Aix-Marseille University, INSERM U1068, CNRS UMR7258, Institute Paoli-Calmettes, Marseille, France; 2 Present Address: Department of Medical Oncology, Institut Paoli-Calmettes, Marseille, France; 3 Therapeutic Innovation Center, Federal University of Pernambuco, Recife, Brazil

**Keywords:** cancer-associated fibroblasts, Tumor-associated macrophages, FOLFIRINOX, Pancreatic cancer, Chemoresistance, Intercellular communication

## Abstract

**Background:**

Pancreatic ductal adenocarcinoma (PDAC) remains a clinically challenging cancer, mainly due to limited therapeutic options and the presence of a highly prominent tumor microenvironment (TME), facilitating tumor progression. The TME is predominated by heterogeneous populations of cancer-associated fibroblasts (CAFs) and tumor associated macrophages (TAMs), in constant communication with each other and with tumor cells, influencing many tumoral abilities such as therapeutic resistance. However how the crosstalk between CAFs and macrophages evolves following chemotherapeutic treatment remains poorly understood, limiting our capacity to halt therapeutic resistance.

**Methods:**

We combined biological characterization of macrophages indirectly cocultured with human PDAC CAFs, under FOLFIRINOX treatment, with mRNAseq analyses of such macrophages and evaluated the relevance of the specific gene expression signature in a large series of primary PDAC patients to search for correlation with overall survival (OS) after FOLFIRINOX chemotherapy.

**Results:**

Firstly, we demonstrated that CAFs polarize naïve and M1 macrophages towards an M2-like phenotype with a specific increase of CD200R and CD209 M2 markers. Then, we demonstrated that CAFs counteract the pro-inflammatory phenotype induced by the FOLFIRINOX on Macrophages. Indeed, we highlighted that, under FOLFIRINOX, CAFs limit the FOLFIRINOX-induced cell death of macrophages and further reinforce their M2 phenotype as well as their immunosuppressive impact through specific chemokines production. Finally, we revealed that under FOLFIRINOX CAFs drive a specific macrophage gene expression signature involving *SELENOP* and *GOS2* that correlates with shortened OS in FOLFIRINOX-treated PDAC patients.

**Conclusion:**

Our study provides insight into the complex interactions between TME cells under FOLFIRINOX treatment. It suggests potential novel candidates that could be used as therapeutic targets in combination with FOLFIRINOX to prevent and alleviate TME influx on therapeutic resistance as well as biomarkers to predict FOLFIRINOX response in PDAC patients.

Video Abstract

**Supplementary Information:**

The online version contains supplementary material available at 10.1186/s12964-023-01388-7.

## Background

Representing the most common malignancy of the pancreas, pancreatic ductal adenocarcinoma (PDAC) is a lethal and particularly aggressive cancer presenting a five-year survival rate of less than 9% [1]. Many factors contribute to this dismal prognosis including tumor stage at diagnosis, with the majority of patients presenting locally advanced disease or metastases, the presence of a highly dense, fibrotic and immunosuppressive tumor microenvironment (TME), and limited therapeutic options [2]. Due to the presence of advanced disease, most patients are ineligible for surgical resection which remains, to this date, the only curative treatment [3]. In comparison with other solid cancers, the development of PDAC treatments, including targeted therapies or immunotherapies, remains in its infancy with few combinatory treatments available, and no alternatives but chemotherapy as first-line therapy [4]. Indeed, the therapeutic armamentarium was limited for a long time to Gemcitabine, the gold-standard for PDAC treatment, but was recently enriched by the FOLFIRINOX, a combination treatment regimen of three different chemotherapeutic agents showing superior benefits, albeit with higher incidence of adverse effects [5, 6].

Despite relatively greater survival benefit, resistance to Gemcitabine and FOLFIRINOX, resulting in disease relapse, remains a major barrier to improve patient outcomes [5]. The uniquely dense TME of PDAC has been identified as a foremost contributor to intrinsic and acquired chemoresistance mechanisms through its physical characteristics disrupting drug delivery, metabolic modulation of drugs, and impairment of their actions within the tumor. The PDAC TME is composed of numerous cell types, predominantly cancer-associated fibroblasts (CAFs) and tumor-associated macrophages (TAMs), and acellular component, the extracellular matrix (ECM) forming the desmoplastic reaction [7]. CAFs are mainly responsible for the deposition and remodeling of the ECM, which acts as a barrier to therapies and anti-tumoral immune cells and also directly contributes to tumor cell proliferation, epithelial-mesenchymal transition, and dissemination [8]. Several subtypes of CAFs have been recently identified revealing a vast inter- and intra-patient heterogeneity [9]. Macrophages have been long-defined to be in different cell states, from the classically-activated, pro-inflammatory M1 phenotype to the alternatively-activated, anti-inflammatory M2 phenotype. TAM populations are proportionally enriched in M2 macrophages, promoting cell proliferation and facilitating immune escape by excluding or inactivating anti-tumoral immune cell types [10]. However, this binary classification is being challenged as recent evidence shows a continuum of macrophages states between M1 and M2 phenotypes depending on the signals received from tumor and TME cells.

Within the PDAC TME, CAFs and TAMs are in constant communication with each other to facilitate tumor progression, aggressiveness, and therapy resistance, which has become a subject of interest. Recently acquired evidence demonstrated highly proliferative CAFs were associated with high macrophage infiltrates and poor overall survival of patients [11]. Interestingly, CAFs promote immunosuppressive macrophage accumulation [12] of TAM phenotypes [13]. Thus, through mutual communication, CAFs and TAMs may together orchestrate the pro-tumoral features of the TME. However, the mechanisms by which this cross-communication is mediated in the presence of active treatment compounds, and its implication in therapeutic resistance remain to be understood.

Consequently, we sought to explore the intercellular communication between CAFs and TAMs in PDAC in the context of FOLFIRINOX treatment to better understand the cellular consequences of this crosstalk and its potential influence on therapeutic resistance. We hypothesized that FOLFIRINOX modifies CAF-mediated reprogramming of TAMs in PDAC. We modelled this intercellular communication by indirect cocultures of human PDAC CAFs with healthy monocyte-derived macrophages, subjected to FOLFIRINOX treatment. Resulting macrophage phenotypes and functional activities as well as transcriptomic profiles were investigated. Primarily, we revealed that the CAFs-driven polarization of macrophages to a M2-like phenotype is further reinforced under FOLFIRINOX treatment. Moreover, secretion by the resulting macrophages of immunosuppressive anti-inflammatory cytokines and chemokines following FOLFIRINOX treatment were increased by CAFs, while protecting them from FOLFIRINOX-induced cell death. Interestingly, transcriptomic profiling of macrophages revealed several genes that were differentially modulated in expression by CAFs in the presence of FOLFIRINOX treatment, highlighting a specific gene signature that correlates with survival in FOLFIRINOX-treated PDAC patients. Overall, our study reveals the impact of CAFs on macrophages phenotype and activity under chemotherapy context, promoting the pro-tumoral activity of macrophages and highlights potential, novel therapeutic targets that could be used to alleviate chemoresistance mechanisms favored by the PDAC TME.

## Methods

### Cell culture

#### Isolation of monocytes and macrophage differentiation

Healthy donor blood, in concentrated buffy coats, was obtained from the French Blood Establishment (Marseille, France). Peripheral blood mononuclear cells (PBMC) were isolated using the Ficoll-Paque density gradient method. Briefly, buffy coats were mixed with RPMI 1640 medium (Gibco) supplemented with 10% FBS and 1% antibiotic-antimycotic and centrifuged at 400 x g for 40 minutes (with 0 acceleration and deceleration) with Ficoll-Paque (Cytvia) to obtain a ring of PBMCs. This ring was isolated, incubated for 10 minutes in 1x red blood cell lysis buffer (eBioscience) and washed in 1X PBS 4 times before cell counting. CD14+ monocytes were isolated from PBMCs using magnetic labeling and sorting with human CD14 microbeads (Miltenyi Biotec) by positive selection following the manufacturer’s protocol and the autoMacs pro separator (Miltenyi Biotec). Purity of isolated monocytes was verified using flow cytometry staining and analysis of CD14 marker expression. Monocytes were plated at a density of 135,000 cells/cm^2^ and treated with 40 ng/mL of recombinant human macrophage colony stimulating factor (M-CSF, Miltenyi Biotec). Following five days of incubation, cell culture medium was changed, supplemented with either lipopolysaccharide (LPS) (10 ng/mL) and IFNy (50 ng/mL) (Peprotech) (to polarize towards M1 macrophages) or IL-4 and IL-13 (50 ng/mL) (Peprotech) (to polarize towards M2 macrophages), and incubated for 48 h. Cocultures were established with immortalized PDAC CAFs at a 1:1 ratio where M0, M1, or M2 macrophages were plated in 12-well plates and CAFs were added to Transwell inserts with 0.4-μm pores (Corning). Cocultures were either left untreated or treated with FOLFIRINOX (15uM) for 48 h before biological readouts were performed.

#### Isolation and culture of primary PDAC cancer-associated fibroblasts (CAFs)

Human primary CAFs were obtained as previously described (Leca et al., 2016). Briefly, the tumors were cut into small pieces of 1 mm3 using a razor blade. The tissue pieces were dissociated using the Tumor Dissociation Kit (Miltenyi Biotec) according to the manufacturer’s recommendations. Cells were then resuspended, passed through a cell strainer (100 μM), and finally plated. Primary CAF features were verified by flow cytometry with a positive α-SMA and FAP staining. Immortalized CAFs were generated from primary CAFs of limited passage via retrovirus-mediated expression of human telomerase reverse transcriptase (hTERT). All CAFs were cultured in DMEM F-12 medium (Gibco) supplemented with 10% fetal bovine serum, 1% L-glutamine (Gibco), 1% antibiotic-antimycotic, and 0.5% sodium pyruvate (Gibco). Cells were detached and passaged using StemPro accutase cell dissociation reagent (Gibco).

All cultured cells were tested for mycoplasma contamination using MycoAlert Mycoplasma Detection Kit (Lonza).

### Flow cytometry

Cells were detached and washed once with 1x PBS and resuspended in FACs buffer (0.5% BSA, 2 mM EDTA in PBS). Staining with extracellular fluorochrome coupled-antibodies was carried out in 100uL of FACs buffer for 20 minutes in the dark at 4 °C. Antibodies used for macrophage phenotyping were: CD80 (BV605 BD Biosciences), CD83 (BV650, BD Biosciences), CD86 (BV786, BD Biosciences), CD200R (PE, Biolegend), CD206 (Alexa-Fluor647, BD Biosciences), CD209 (BV421, BD Biosciences). Viability staining was performed by adding one drop of Sytox green flow reagent (ThermoFisher Scientific) to samples prior to analysis. Compensation was performed using either Ultracomp eBeads plus compensation beads (Invitrogen). All samples were rewashed in FACs buffer once before being analyzed using a BD LSR-Fortessa X20 cell analyzer. Data were analyzed using the FlowJo software (BD Biosciences).

### Sartorius/Incucyte live imaging

#### Viability assay

To measure viability of macrophages, live cell imaging was performed with the Incucyte S3 live-cell analysis system (Sartorius). Following coculture with CAFs and/or treatment with FOLFIRINOX for 48-hours, macrophages at 30% confluency were incubated with 250 nM of Incucyte® cytotox green dye (Sartorius). The green dye enters cells upon compromise of plasma membrane integrity and binding to DNA. Images were taken with the following parameters: two images per well (3 wells per condition), 10x magnification, 350 ms acquisition time, every 2 hours for a duration of 3 days. Integrated adherent cell-by-cell analysis was performed to calculate total green integrated intensity, representative of dying or dead cells, which was normalized to cell confluency and used to calculate viability of macrophages.

#### Phagocytosis assay

To measure phagocytic capacity of macrophages, live cell imaging was performed with the same system as described above. Following coculture with CAFs and/or treatment with FOLFIRINOX for 48-hours, macrophages at 30% confluency were incubated with 10 μg of IncuCyte® pHrodo® green *E. coli* Bioparticles®. Phagocytosis is measured by detection of fluorescence in cells when *E. coli* bioparticles are engulfed by macrophages and enter their acidic phagosomes, leading to an increase in fluorescence. Images were taken with the following parameters: two images per well (3 wells per condition), 10x magnification, 400 ms acquisition time, every 15 minutes for a duration of 3 hours. Integrated adherent cell-by-cell analysis was performed to calculate total green integrated intensity, representative of phagocytosing cells, which was normalized to cell confluency and used to calculate phagocytic capacity of macrophages.

### Multiplex ELISA (Luminex)

For multiplex ELISA analysis of conditioned medium of monocyte-derived macrophages in various conditions, conditioned medium of macrophages was collected and centrifuged at 250×g for 5 minutes to remove cells and debris. Conditioned media were aliquoted and stored at − 80 °C until use. They were thawed on ice and analyte expression was assessed using a custom Human Procartaplex mix-and-match 32-plex panel following the manufacturer’s protocol (ThermoFisher). The panel included: BLC (CXCL13), CD40L, Eotaxin-2 (CCL24), Eotaxin-3 (CCL26), Fractalkine (CX3CL1), GRO alpha (CXCL1), IFN gamma, IL-1 beta, IL-1RA, IL-6, IL-8 (CXCL8), IL-10, IL-13, IL-15, IL-21, IL-23, IP-10 (CXCL10), MCP-1 (CCL2), MCP-3 (CCL7), MCP-4 (CCL13), MDC, MIG (CXCL9), MIP-1 alpha (CCL3), MIP-3 alpha (CCL20), MMP-1, RANKL, RANTES (CCL5), SDF-1 alpha, TARC (CCL17), TNF alpha, VEGF-A, VEGF-D. Optical density measurement of plates was performed using the Bioplex 200 (BioRad). Analytes with measurements exceeding or below the values of the standard curve were excluded from analysis.

### RNA sequencing and analysis

Macrophages from all conditions were detached as previously described and RNA was extracted using the RNeasy mini kit (Qiagen). Quality of RNA was analyzed using the RNA 6000 Nano kit (Agilent) and read on the 2100 Bioanalyzer instrument (Agilent). Libraries are prepared with NEBNext Ultra II Directional RNA Library Prep Kit for Illumina protocol according supplier recommendations. Briefly the key stages of this protocol are successively, the purification of PolyA containing mRNA molecules using poly-T oligo attached magnetic beads from 100 ng total RNA (with the Magnetic mRNA Isolation Kit from NEB), a fragmentation using divalent cations under elevated temperature to obtain approximately 300 bp fragments, double strand cDNA synthesis and finally Illumina adapters ligation and cDNA library amplification by PCR for sequencing. Sequencing is then carried out on Paired End 100b reads on Illumina NovaSeq6000. Image analysis and base calling is performed using Illumina Real Time Analysis with default parameters. Sequences were quality checked using FASTQC. Low quality bases (Phred quality score less than 30) were filtered out and TrueSeq Adapters were trimmed using trimmomatic [14]. Reads were mapped to hg38 using subread-align (v1.5.0) [15] with default parameters. The aligned reads were summarized at the gene-level using featureCounts [15] counts were normalized by the size of corresponding library (DESeq2, estimateSizeFactors function) and finally, differentially expressed genes (DEG) analysis was performed using DESeq2 package with default parameters. Genes were considered as DEG if they achieved a false discovery rate of 5% or less. Gene annotation was carried out using *Homo sapiens* (org.Hs.eg.db) AnnotationDbi from R/Bioconductor [16]. Enriched Ontology clusters was carried out using Metascape.org. Briefly, all statistically enriched terms were identified (GO/KEGG terms, canonical pathways), then accumulative hypergeometric *p*-values and enrichment factors were calculated and used for filtering. Remaining significant terms were then hierarchically clustered into a tree based on Kappa-statistical similarities among their gene memberships. Finally, 0.3 kappa score was applied as the threshold to cast the tree into term clusters.

### Analysis of gene expression profiles of clinical PDAC samples

We gathered clinicopathological and gene expression data of clinical PDAC samples from 16 publicly available data sets (Supp. Table 1) collected from ArrayExpress, EGA, National Center for Biotechnology Information (NCBI)/Genbank GEO, and TCGA databases. Expression profiles had been generated using DNA microarrays (Affymetrix, Agilent, Illumina) and RNA-seq (Illumina). The final pooled data set contained 938 primary PDAC samples. The pre-analytic processing of data was done as previously described [17]. Then, the log_2_-transformed tumor expression levels of each of the 10 genes of interest (*CCDC152, GO*S*2, GPR155, LAD1, METTL27, NLRP1, SELENOP, SHE, SLC40A1* and *SYNPO)* were analysed as discrete values by using the median expression level as cut-off, thus defining two tumor classes thereafter designated “high” and “low”.

### Statistical analyses

Statistical analyses of molecular experiments were performed on GraphPad Prism 8 using paired non-parametric student’s t-tests. Statistical analyses of public gene expression data from clinical samples were done using R software. Overall survival (OS) was calculated from the date of diagnosis until the date of death from any cause. Follow-up was measured from the date of diagnosis to the date of last news for alive patients. Survivals were calculated using the Kaplan-Meier method and curves were compared with the log-rank test. Uni- and multivariate prognostic analyses for OS were done using Cox regression analysis (Wald test). The variables submitted to analyses included the tumor classes (“high” and “low”) based on gene expression levels, patients’ age (> 60 years vs ≤60 years) and gender (male vs female), and pathological American Joint Committee on Cancer (AJCC) stage (2 vs 1, and 3 vs 1). Multivariate analysis incorporated the variables with a *p*-value inferior to 5% in univariate analysis. The likelihood ratio (LR) tests were used to assess the prognostic information provided by each 2-gene model to each 1-gene model, assuming a X2 distribution. Changes in the LR values (LR-ΔX2) measured quantitatively the relative amount of information of one model compared with another. All statistical tests were two-sided at the 5% level of significance. *P-values* < 0.05 were considered statistically significant.

## Results

### Cafs polarize macrophages towards a M2 macrophage phenotype

**Fig. 1:**
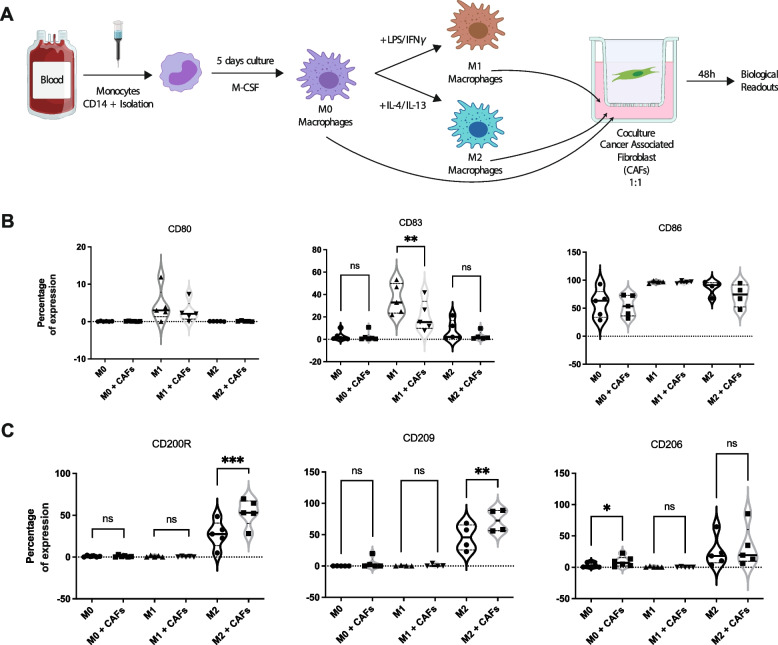
Phenotyping of polarized macrophages M0, M1, and M2 in monoculture or coculture with PDAC CAFs. **A** Schematic depiction of experimental protocol. **B** Expression of M1 markers CD80, CD83 and CD86 in macrophages with or without CAFs. **C** Expression of M2 markers CD200R, CD209, and CD206 in macrophages with or without CAFs. Significant differences in expression with *p*-values < 0.0001 ****, < 0.001 ***, < 0.01 **, and < 0.05 * are indicated

To characterize macrophage polarization and phenotypes under the distant influence of CAFs, cocultures, avoiding cell-cell contact, using Transwell inserts were established between immortalized human PDAC CAFs and M0, M1 and M2 macrophages. Healthy blood donor-derived monocytes were differentiated in culture for five days with recombinant human M-CSF to obtain M0 macrophages, which were further polarized to either M1 macrophages by stimulation with IFNγ and lipopolysaccharide (LPS), or to M2 macrophages by stimulation with interleukins IL-4 and IL-13 for 48 hours. Then, following 48-hours of coculture with CAFs, macrophages were recovered and analyzed by flow cytometry using M1 and M2 macrophage markers (Fig. 1A). Classic polarization states of M1 and M2 macrophages were confirmed where M1 macrophages expressed high levels of CD86 with low and moderate expression of CD80 and CD83 respectively (Fig. 1B, Supp. Fig. 1A). M1 macrophages did not express M2 markers CD209, CD206, and CD200R (Fig. 1C, Supp. Fig. 1B). Inversely, M2 macrophages showed increased expression of M2 markers CD209 and CD200R (Fig. 1, Supp. Fig. 1B) while M1 markers’ expression in M2 polarized macrophages was null for CD80 and CD83 (Fig. 1B, Supp. Fig. 1A). We observed that the addition of CAFs to M1 macrophages resulted in a significant decrease of M1 marker CD83 (Fig. 1B, Supp. Fig. 1A) while it did not modify any M2 markers’ expression (Fig. 1C). Moreover, the expression of CD80, CD83, and CD86 markers was unaffected by the presence of CAFs in M0 and M2 macrophages. However, polarized M2 macrophages demonstrated a further significant increase in expression of M2 markers CD209 and CD200R upon coculture with CAFs (Fig. 1C). Additionally, M0 macrophages also showed a significant increase in CD206 expression with CAFs, suggesting that CAFs were capable of pushing unpolarized macrophages towards a M2 phenotype. Overall, our data demonstrate that macrophages are favoured towards a M2 phenotype under the influence of PDAC CAFs.

### CAFs reinforce M2 macrophage polarization under FOLFIRINOX treatment

**Fig. 2:**
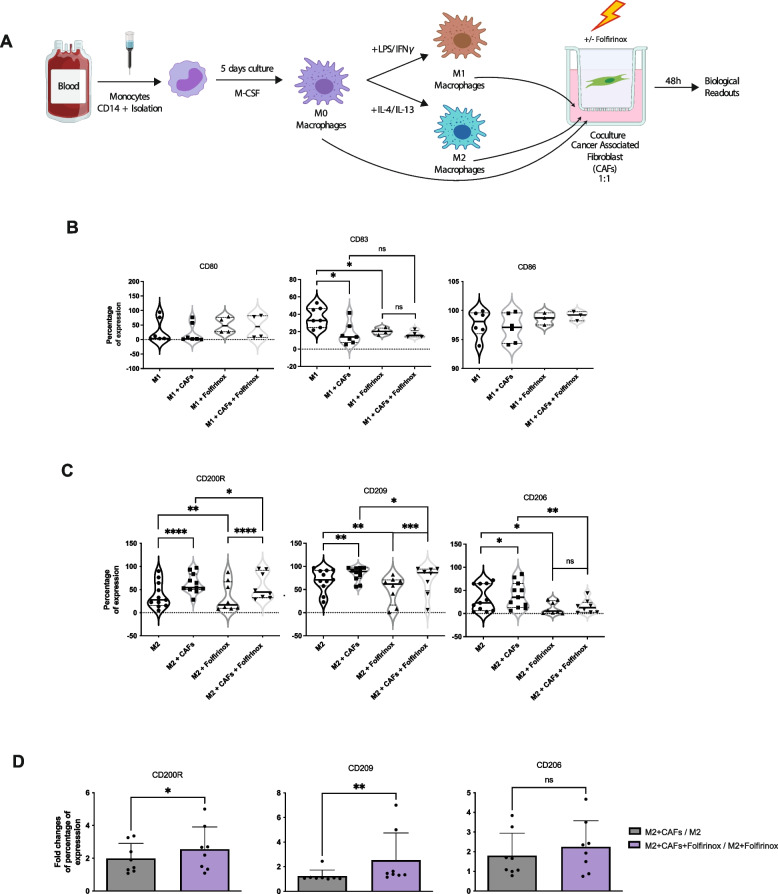
Phenotyping of polarized M1 and M2 macrophages cocultured with PDAC CAFs under FOLFIRINOX treatment. **A** Schematic depiction of experimental protocol. **B** M1 macrophage markers, CD80, CD83 and CD86, expression in M1 macrophages with or without CAFs coculture and with or without FOLFIRINOX treatment. **C** M2 macrophage markers, CD200R, CD209, and CD206, expression in M2 macrophages with or without CAFs coculture and with or without FOLFIRINOX treatment. **D** Fold change of percentage of expression of CD200R, CD209, and CD206 between M2 macrophages cocultured with CAFs vs. monoculture M2 macrophages, and M2 macrophages treated with FOLFIRINOX and cocultured with CAFs vs. M2 macrophages treated with FOLFIRINOX in monoculture. Significant differences in expression with p-values < 0.0001 ****, < 0.001 ***, < 0.01 **, and < 0.05 * are indicated

In order to understand the potential chemoprotective and polarizing effects of CAFs on macrophages, M1 and M2 macrophages were placed in coculture with CAFs and were subject to FOLFIRINOX treatment at a concentration of 15uM of each drug composite: 5′-fluorouracil, irinotecan, and oxaliplatin for 48 hours. The conditions studied are as follows: M1 or M2 macrophages, M1 or M2 macrophages cocultured with CAFs, M1 or M2 macrophages treated with FOLFIRINOX, and M1 or M2 macrophages cocultured with CAFs and treated with FOLFIRINOX. Following coculture and treatment, macrophages were recovered and analyzed by flow cytometry for M1 and M2 macrophage marker expression (Fig. 2A). For polarized M1 macrophages, CD80 and CD86 expression was unaffected by both FOLFIRINOX treatment and presence of CAFs (Fig. 2B, left and right panels respectively, Supp. Fig. 1C). We confirmed that CD83 expression significantly decreases for M1 macrophages when cocultured with CAFs (Fig. 2B, middle panel, Supp. Fig. 1C) as seen above (Fig. 1B). FOLFIRINOX treatment also resulted in a significant decrease in CD83 expression. However, in the presence of CAFs, the already reduced expression of CD83 was not further impacted, suggesting that the presence of CAFs overbears treatment with chemotherapy (Fig. 2B, middle panel, Supp. Fig. 1C). As precedently observed, M2 macrophages expressed higher levels of CD209 and CD200R when cocultured with CAFs (Fig. 2C, Supp. Fig. 1D). FOLFIRINOX treatment resulted in significant decreases of M2 marker expression, CD209, CD206, and CD200R in M2 polarized macrophages where CD206 showed the strongest reduction (Fig. 2C, Supp. Fig. 1D). However, when macrophages were cocultured with CAFs, under FOLFIRINOX treatment, the expression of CD209 and CD200R was significantly increased, revealing that CAFs maintain an M2 phenotype under FOLFIRINOX treatment (Fig. 2C, Supp. Fig. 1D). It’s interesting to note that coculture with CAFs combined with FOLFIRINOX treatment resulted in a stronger increase of M2 marker expression in polarized M2 macrophages as evidenced by the significantly higher fold-changes of two out of the three markers’ expression (CD209 and CD200R), between M2-CAFs-FOLFIRINOX and M2-FOLFIRINOX compared to M2-CAFs and M2 (Fig. 2D). Taken together, these results demonstrate that CAFs promote M2 macrophage polarization and also favor an alternative M2 phenotype under FOLFIRINOX treatment.

### CAFs increase viability and modify activity of polarized M2 macrophages under FOLFIRINOX treatment

**Fig. 3:**
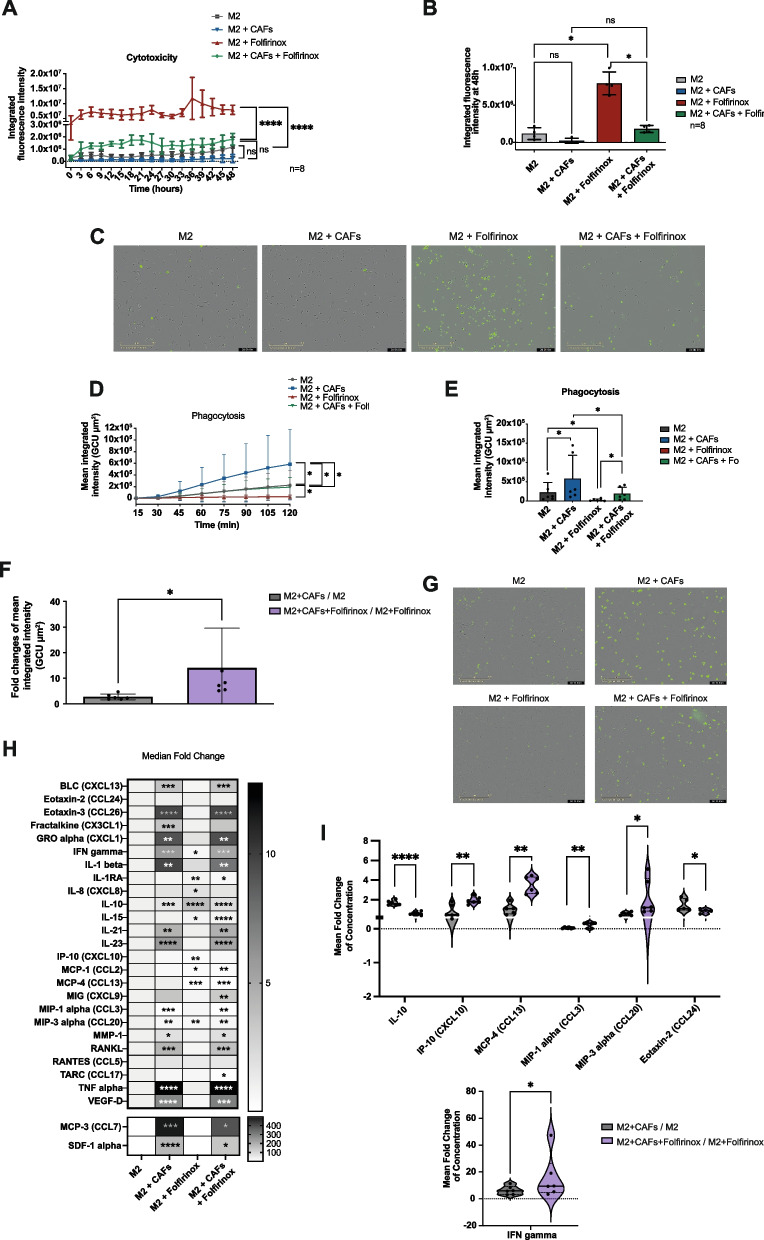
M2 macrophage viability, phagocytosis, and secretory profile in monoculture or coculture with PDAC CAFs under FOLFIRINOX treatment. **A** Expression of dead/dying-cell marker, cytotox green fluorescent protein, in M2 macrophages over 48 hours by Incucyte live-imaging. **B** Integrated green fluorescence intensity of cytotox protein at 48 hours in M2 macrophages. **C** Representative images of cytotox fluorescence in untreated, monoculture M2 macrophages, FOLFIRINOX-treated monoculture M2 macrophages, untreated CAF-cocultured M2 macrophages, and FOLFIRINOX-treated CAF-cocultured M2 macrophages with indicated scale at 2 days. **D** Phagocytosis of green fluorescent E.coli bioparticles measured by integrated green fluorescence intensity in M2 macrophages over 2 hours. **E** Integrated green fluorescence intensity of E.coli bioparticles in M2 macrophages at 2 hours. **F** Comparison of fold changes of phagocytosis capacity between M2 macrophages cocultured with CAFs vs. M2 macrophages in monoculture and M2 macrophages cocultured with CAFs treated with FOLFIRINOX vs. M2 macrophages cocultured with CAFs untreated. **G** Representative images of fluorescent E.coli bioparticle uptake in M2 macrophages in conditions as described in (C) at 105 minutes. **H** Heat map of fold-change of concentration (pg/mL) of 27 cytokines/chemokines in M2 macrophages measured using multiplex ELISA. **I** Comparison of fold changes of cytokine and chemokine concentrations between M2 macrophages cocultures with CAFs vs. M2 macrophages in monoculture and M2 macrophages cocultured with CAFs treated with FOLFIRINOX vs. M2 macrophages cocultured with CAFs untreated. Significant differences in expression with p-values < 0.0001 ****, < 0.001 ***, < 0.01 **, and < 0.05 * are indicated

To observe whether CAFs could protect M2 macrophages from FOLFIRINOX-induced cell death, viability assays using cytotox green dead-cell fluorescent marking dye were performed on macrophages from mono- or CAFs co-cultures coupled with or without FOLFIRINOX treatment. As expected, FOLFIRINOX treatment resulted in a significant increase in cytox fluorescent marking, representative of decreased viability, of macrophages over a 48 hour period. Macrophages treated with FOLFIRINOX and cocultured with CAFs showed a significant 5-fold reduction of green fluorescence intensity compared to FOLFIRINOX-treated macrophages, demonstrating that CAFs protect macrophages from chemotherapy-induced cell death (Fig. 3A-C).

We next sought to investigate the functional phagocytic and secretory capacities of these macrophages. A phagocytic assay was performed on M2 macrophages in mono- or coculture with CAFs, with or without FOLFIRINOX treatment, by incubation with fluorescent *E. coli* bioparticles and live-cell imaging analysis over a period of 120 minutes. Macrophages in coculture with CAFs showed a significant increase in phagocytic activity compared to monoculture macrophages. FOLFIRINOX treatment significantly reduced phagocytic activity of macrophages which was recovered by the addition of CAFs, demonstrating yet again the chemoprotective effect of CAFs on macrophages (Fig. 3D-F). Interestingly, CAFs had a greater impact on inducing macrophage phagocytosis in the presence of FOLFIRINOX, as evidenced by the significantly higher fold-changes between the two comparisons (Fig. 3G). *S*ecretory profiles of macrophages under these same conditions were analyzed using a custom 32-plex Luminex assay (Fig. 3H). FOLFIRINOX treatment resulted in a significant decreased secretion of cytokine and chemokine including IFNg, IL-1RA, IL-15, IP-10 (CXCL10), MCP-1 (CCL2), MCP-4 (CCL13), and MIP-3 alpha (CCL20) by M2 macrophages. Particularly, IL-8 and IL-10 secretion was significantly increased upon FOLFIRINOX treatment by M2 macrophages, where only IL-8 upregulation was specific to FOLFIRINOX treatment (Fig. 3H). The presence of CAFs, regardless of FOLFIRINOX treatment, commonly led to the upregulation of 13 secreted proteins including CXCL13, CCL26, CXCL1, IL-1b, IL-21, IL-23, CCL3, MMP-1, RANKL, TNFa, VEGF-D, CCL7, and SDF-1a. Additionally, CCL17 was significantly downregulated by presence of CAFs specifically under FOLFIRINOX treatment respectively. To investigate how CAFs differently impact the secretion profile of M2 macrophages under chemotherapy treatment, significantly different fold-changes of these differentially regulated cytokines and chemokines between addition of CAFs with or without FOLFIRINOX treatment were used to select proteins. This filter reduced the 13-secreted protein list to 7 proteins. In the presence of FOLFIRINOX, CAFs were less capable of upregulating expression of IL-10 and CCL24, whereas they further boosted expression of CXCL10, CCL13, CCL3, CCL20, and IFNy compared to without FOLFIRINOX treatment (Fig. 3I and Supp. Figure 2A). These upregulated proteins thus represent the impact of CAFs on the chemo-secretory profile of M2 macrophages.

### The presence of CAFs leads to macrophage transriptomic reprogramming under FOLFIRINOX treatment

**Fig. 4:**
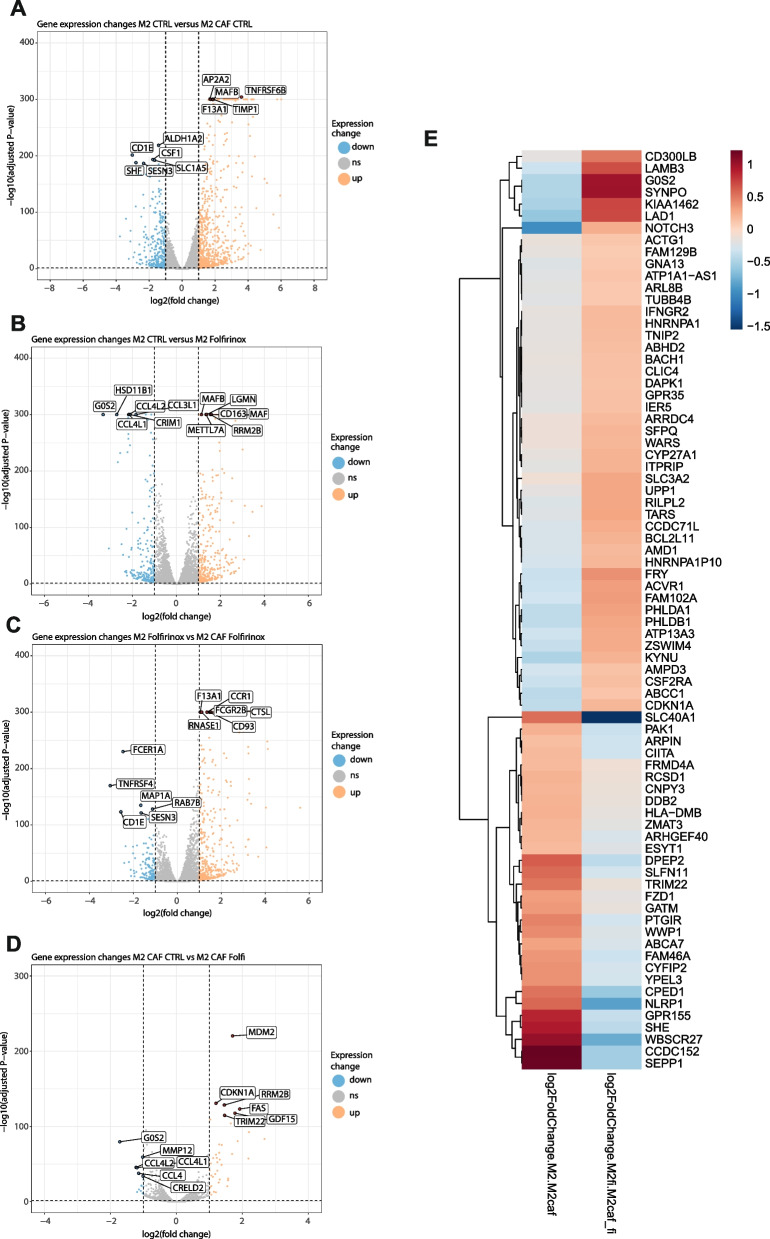
RNA sequencing analysis of M2 macrophages cocultured with PDAC CAFs and/or treated with FOLFIRINOX. Volcano plots of differential gene expression between (**A**) M2 macrophages and M2 macrophages cocultured with CAFs (**B**) M2 macrophages treated with FOLFIRINOX vs. untreated (**C**) M2 macrophages treated with FOLFIRINOX in monoculture vs. in coculture with CAFs (**D**) M2 macrophages in cocultures with CAFs treated and untreated with FOLFIRINOX. Genes were considered significantly differentially regulated if p-value < 0.05 and log_2_fold-change > 1. **E** Heat map of group “0” showing log2-fold changes in M2 macrophages vs. M2 macrophages cocultured with CAFs and log2-fold changes in M2 macrophages treated with FOLFIRINOX in mono- or cocultures with CAFs

Thus far, we identified the phenotypic and functional modifications driven by CAFs on M2 macrophages under FOLFIRINOX treatment. To further characterize the impact of CAFs on macrophages under FOLFIRINOX treatment, we performed gene expression profiling by RNA sequencing. M2 macrophages were subjected to the various cultures as previously described before performing RNA extraction followed by sequencing. Gene expression was considered to be significantly deregulated based on a log_2_-fold change greater or less than 1 and a *p*-value below 0.05. Coculture of M2 macrophages with CAFs resulted in increased expression of 827 genes and decreased expression of 398 genes in M2 macrophages (Fig. 4A and Supp. Figure 3A). Of note, addition of CAFs led to strong upregulation of several chemokines including CCL18, CCL23, CCL24, and CCL26 as observed in our Luminex analysis (Fig. 3H) and of phagocytosis receptors and related proteins FCGR1A, FCGR2A and FCGR3A, CD93, and MYO7A, confirming data from our phagocytosis assay (Fig. 3D). FOLFIRINOX treatment resulted in 337 upregulated and 231 downregulated genes in M2 macrophages (Fig. 4B and Supp. Figure 3B). The addition of CAFs to macrophages treated with FOLFIRINOX resulted in the overexpression of 440 genes compared to reduced expression of 141 genes (Fig. 4C and Supp. Figure 3C). Finally, comparison of CAFs-cocultured macrophages with FOLFIRINOX with untreated CAFs cocultures resulted in lesser changes in gene expression with only 40 and 15 upregulated and downregulated genes respectively, demonstrating that CAFs have a greater influence on macrophage gene expression profiles that chemotherapy treatment cannot significantly overcome (Fig. 4D and Supp. Figure 3D).

To delineate the specific impact of CAFs on M2 macrophages under FOLFIRINOX treatment, we performed a comparison allowing the identification of gene expression modifications in macrophages not only by the presence of CAFs but the specific influence of CAFs under FOLFIRINOX treatment. Thus, we compared the differential regulation of genes between “monoculture M2 macrophages *vs*. M2 macrophages cocultured with CAFs” and “M2 macrophages treated with FOLFIRINOX *vs*. M2 macrophages cocultured with CAFs and treated with FOLFIRINOX” (Fig. 4E). We denoted three different groups based on gene expression up- and downregulation and their amplitudes. Group “0” identified genes that were differentially expressed in each comparison but with the opposite trends, being upregulated by the addition of CAFs in control macrophages but downregulated with the addition of CAFs under FOLFIRINOX treatment or vice versa. Group “1” identified genes that were consistently upregulated in M2 macrophages following the addition of CAFs but with different amplitude under FOLFIRINOX treatment. Group “-1” identified genes that were consistently downregulated in M2 macrophages following the addition of CAFs but with different amplitude under FOLFIRINOX treatment. Overall, we demonstrated the differential impact of CAFs on macrophages gene expression reprogramming dependent on FOLFIRINOX and highlighted novel key genes and enriched ontology clusters (Supp. Figure 4A) that may mediate the pro-tumoral effects of M2 macrophages in communication with CAFs in the specific context of FOLFIRINOX treatment.

### Selenop/GOS2 combined expression in clinical samples represents a valuable clinical measurement of CAFs-driven macrophages impact on PDAC patients response to FOLFIRINOX

**Fig. 5:**
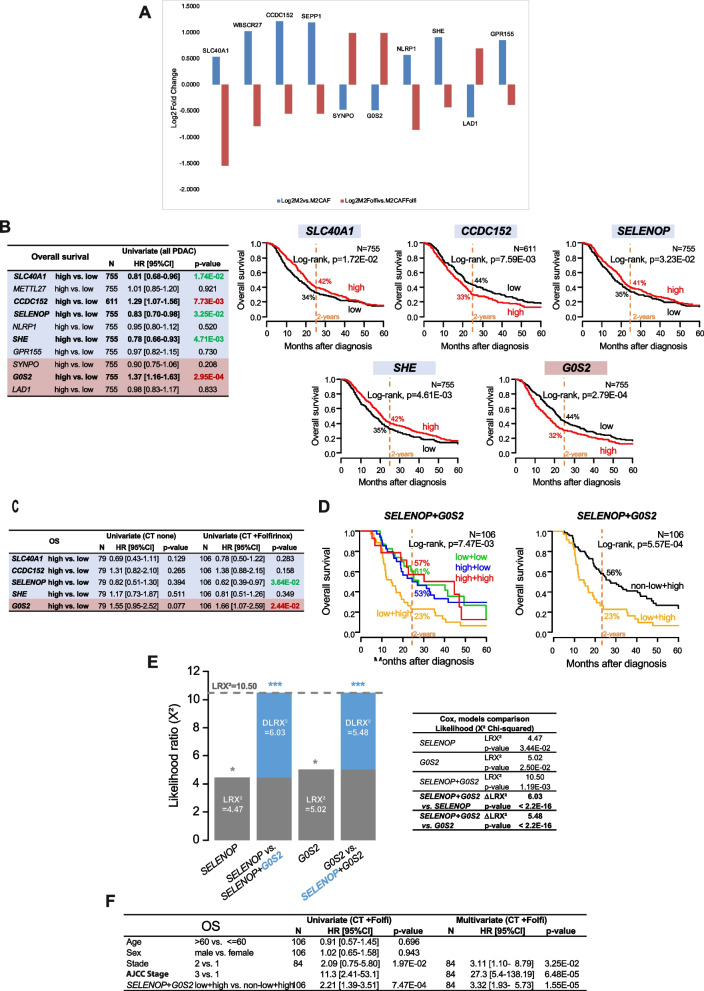
Analysis in clinical PDAC samples of expression of 10 under genes differentially expressed in macrophage under impact of CAFs and FOLFIRINOX treatment. **A** Bar graph indicating the log_2_-fold change of the top 10 genes from group “0”. **B** Left: Univariate analyses for OS in the whole population for the 10 genes. Right: Kaplan-Meier OS curves according to “high” and “low” mRNA expression in the whole population for the five significant genes. **C** Univariate analysis for OS and the five significant genes in two sub-populations: patients without any post-operative chemotherapy (“CT none”) and patients with delivery of post-operative FOLFIRINOX chemotherapy (“CT + FOLFIRINOX”). **D** Kaplan-Meier OS curves according to combined “high” and “low” mRNA expression of *SELENOP* and *GOS2* in the sub-population treated by post-operative FOLFIRINOX chemotherapy. **E** Comparison of prognostic information (OS) of the 2-gene model versus each 1-gene models in the sub-population treated by post-operative FOLFIRINOX chemotherapy. **F** Multivariate analysis for OS in the sub-population treated by post-operative FOLFIRINOX chemotherapy

We considered the gene group “0” as being of particular interest since it highlighted genes with expression patterns that were completely switched in M2 macrophages under the influence of CAFs in a FOLFIRINOX-dependent manner. We decided to focus our attention on the 10 genes with the highest and the most significant dysregulation: *SLC40A1, METTL27, CCDC152, SELENOP, NLRP1, SHE, GPR155, SYNPO, GOS2* and *LAD1* (Fig. 5A). We analysed their expression in our pooled database of tumor gene expression profiles from 938 patients with primary PDAC (Supp. Table 1). Expression was heterogeneous for all genes and ranged from < 4 intervals (NLRP1) to > 7 intervals (GOS2) in log_2_ scale (Supp. Figure 4B), allowing to search for correlations with clinical data. Overall survival (OS) data were available for 755 patients: 229 (30%) remained alive during a median follow-up of 17 months (range, 1–56), 526 (70%) died, and the 5-year OS was 14% (95%CI, 11–18). In univariate analysis for OS, five genes displayed expression level significantly associated with OS (Fig. 5B). Higher tumor expression was associated with shorter OS for 2 genes: *CCDC152* (HR = 1.29 95%CI 1.07–1.56; Wald test) and *GOS2* (HR = 1.37 95%CI 1.16–1.63), whereas higher expression was associated with longer OS for 3 genes: *SLC40A1* (HR = 0.81 95%CI 0.68–0.96), *SELENOP* (HR = 0.83 95%CI 0.70–0.98), and SHE (HR = 0.78 95%CI 0.66–0.93). Expression of *METTL27, NLRP1, GPR155, SYNPO*, and *LAD1* was not associated with OS. The Kaplan-Meier curves for the 5 significant genes are shown in Fig. 5B. We then analysed the prognostic value of expression of these 5 genes accroing to the delivery of FOLFIRINOX post-operative chemotherapy. As shown in Fig. 5C, no gene was associated with OS in the 79 patients not treated with chemotherapy. By contrast, 2 genes, *SELENOP* (HR = 0.62 95%CI 0.39–0.97) and *GOS2* (HR = 1.66 95%CI 1.07–2.59), were associated with OS in the 106 patients treated with FOLFIRINOX. Given their opposite prognostic value, we searched for an eventual prognostic complementarity between both genes in this patient population. Figure 5D shows Kaplan-Meier curves in the four patients classes defined by combined tumor expression of *SELENOP* and *GOS2*: patients with SELENOP-low/GOS2-low, SELENOP-high/GOS2-low, and SELENOP-high/GOS2-high statutes showed similar OS and were thus merged in a single class (non-SELENOP-low/GOS2-high), which displayed 56% 2-year OS (95%CI 46–70). By contrast, the patients with SELENOP-low/GOS2-high expression displayed worse outcome with 23% 2-year OS (95%CI 12–44, *p* = 5.57E-04, log-rank test). Such prognostic complementarity between expression status of *SELENOP* and *GOS2* was tested using the likelihood ratio (LR) test (Fig. 5E): *GOS2* expression status added prognostic information to that provided by *SELENOP* expression status (ΔLR-X2 = 6.03, *p* < 2.2E-16), and vice-versa (ΔLR-X2 = 5.48, p < 2.2E-16), suggesting a cooperation between these two genes in prognostic term after FOLFIRINOX post-operative chemotherapy. Finally, multivariate analysis for OS including the tumor stage showed independent prognostic value for the combined tumor expression of *SELENOP* and *GOS2* (*p* = 1.55E-05, Wald test; Fig. 5F).

## Discussion

The substantial proportion of the TME and the predominating cell types within, CAFs and TAMs, are undeniably major hallmarks of PDAC and thus actors of tumor progression and therapy resistance. These two cell types protect PDAC tumor cells from anti-tumoral immune cell activities and chemotherapies, concomitantly promoting their growth and dissemination. The communicative network created between CAFs, TAMs and tumor cells is crucial in propagating these effects throughout tumor evolution, as well as under presence of chemotherapeutic agents, but the mechanisms of this crosstalk remain elusive. In this study, we highlighted the differential impact of CAFs on macrophage phenotype and activity in the presence of the chemotherapy regimen, FOLFIRINOX. We have demonstrated that CAFs lead to the formation of anti-inflammatory M2 macrophages from unpolarized macrophages and boost already polarized macrophages further towards this phenotype. Under FOLFIRINOX treatment, M2 macrophages polarization is weakened but recovered by the presence of CAFs. Additionally, we showed that CAFs protect macrophages from FOLFIRINOX-induced cell death, restore their phagocytic capacity as well as their secretory profile of immunosuppressive and anti-inflammatory cytokines and chemokines. Through transcriptomic analysis, we demonstrated the specific impact of CAFs on macrophage gene expression under FOLFIRINOX, highlighting novel potential mechanisms of chemoresistance mediated by the TME as well as new biomarkers predicting FOLFIRINOX-response in PDAC patients.

M2-macrophage polarization is associated with worse overall survival in PDAC and long-term PDAC survivors have significantly reduced levels of M2 macrophages, inversely associated with a T-cell inflamed tumor type [18–21]. As part of the front-line of innate immunity, M2 macrophages mainly mediate their pro-tumoral functions by facilitating immune escape through aggravation of immune checkpoint expression, secretion of immunosuppressive cytokines, and induction of regulatory T-cells. Although polarization of macrophages in the PDAC TME has become an extensive area of investigation, the majority of these studies focus on tumor-mediated polarization. However, CAFs outnumber tumor cells in the tumor bulk and are in close contact with macrophages and infiltrating monocytes in the TME, representing key potential modulators of macrophage activity. We showed that CAFs polarized macrophages towards a M2-phenotype as evidenced by classic M2 macrophage markers’ expression (CD200R and CD209), confirming a previous study that had demonstrated the effect of CAFs on monocyte-to-macrophage differentiation through M-CSF and reactive oxygen species (ROS) production [22]. By treatment with FOLFIRINOX, the M2 phenotype was reduced but extensively recovered by the addition of CAFs, demonstrating that CAFs can protect macrophages from FOLFIRINOX in maintaining their M2 phenotype. Interestingly, the presence of CAFs led to significantly higher increases in M2 macrophage markers expression CD200R and CD209 under FOLFIRINOX treatment than without treatment, showing that CAFs-response to FOLFIRINOX further enhance M2 polarization. It is well-established that CAFs contribute to chemoresistance mechanisms through their interaction with tumor cells. Here, we showed that CAFs may mediate such mechanisms through specific alteration of macrophages under FOLFIRINOX treatment.

We assessed the viability of macrophages under these conditions and demonstrated that although FOLFIRINOX resulted in strong increase of M2 macrophage cell death, in the presence of CAFs this effect was abrogated, revealing that CAFs protect macrophages from FOLFIRINOX-induced cell death. Studies using Gemcitabine have demonstrated that CAFs confer chemoresistance in part by uptake of the drug, rendering it unavailable for tumor cells, by reducing drug transporter protein expression on tumor cells, as well as reducing metabolism of the drug to its active form [23–25]. These mechanisms may potentially also apply to CAF-mediated protection of macrophages from FOLFIRINOX cell death in our model. In line with the augmented expression of M2 markers in macrophages in the presence of CAFs and FOLFIRINOX treatment, we observed the augmentation of a M2 macrophage function; phagocytosis. As they are involved in mitigating inflammatory response and in promoting wound healing, M2 macrophages are more phagocytic than their M1 counterparts with higher CD209 correlating with particle uptake, and are responsible for clearing apoptotic cells [26, 27]. Efferocytosis, the engulfment of apoptotic cells, has been found to induce a tolerogenic phenotype in macrophages [28]. Our findings support these studies demonstrating an increase of phagocytosis associated with high CD209. Indeed, while phagocytic capacity was lost under FOLFIRINOX treatment, CAFs were able to restore macrophage phagocytic activity under FOLFIRINOX treatment. It was found that chemotherapy-induced apoptotic PDAC cell phagocytosis by TAMs led to pro-tumoral and anti-apoptotic signal secretion by TAMs, supporting survival of tumor cells [29]. Thus, increased phagocytosis may be implicated in macrophage-mediated chemoresistance, highlighted in the present study as promoted by CAFs, by a specific response to FOLFIRINOX.

The pro-tumoral functions and regulation of tumor immunity by macrophages can be mediated by the secretion of soluble factors, cytokines and chemokines, in the TME. Our results further confirmed that CAFs were specifically modifying M2 macrophages under FOLFIRINOX treatment, through modulating their secretory profile. More interestingly, CAFs were able to alleviate the decrease in secretion of CCL13 and CCL20 under FOLFIRINOX treatment while decreasing specifically the expression of TARC/CCL17, an effector T-cell chemokine. Among all chemokines boosted by CAFs under FOLFIRINOX treatment, CCL13 had the greatest increase in fold-change of expression. CCL13 is a M2 macrophage-secreted chemokine, an important chemoattractant for monocytes and T-cells, and has been correlated with poor prognosis and tumor progression in ovarian cancer and oral squamous carcinoma, with no information on its involvement in PDAC [30, 31]. Overall, the upregulation of these chemokines in macrophages under the influence of CAFs and FOLFIRINOX treatment may be key in modulation of macrophages under chemotherapy and potential contributors to chemoresistance.

Transcriptomic profiling allowed for further characterization of macrophages under the influence of CAFs and FOLFIRINOX treatment but we sought to focus on the difference in impact of CAFs on macrophage reprogramming when untreated or treated with FOLFIRINOX. To this aim, we analyzed the differences in fold-changes of expression of genes in macrophages, by the influence of CAFs with or without FOLFIRINOX treatment. This analysis revealed three groups of genes that we classified as “-1”, “1”, and “0” corresponding to consistently downregulated genes, upregulated genes, and genes with opposite regulation between the two comparisons respectively. Thus, the differential dysregulation of these genes reveals that CAFs impact specifically macrophages’ response to FOLFIRINOX. Precisely, we found 77 genes within this group that were significantly deregulated in both comparisons with opposite expression profiles. The top ten genes with greatest difference in log_2_-fold changes and listed as follows in order of decreasing difference in log_2_-fold changes: *SLC40A1*, *WBSCR27* (*METLL27*), *CCDC152*, *SELENOP* (*SEPP1*), *SYNPO*, *G0S2*, *NLPR1*, *SHE*, *LAD1*, and *GPR155* have been found to be involved in iron and selenium transport, pyroptosis, actin dynamics, cell motility, adhesion, and transport of growth factors and anti-cancer drugs. We performed clinical correlation analyses of these ten genes in a combined PDAC transcriptomic cohort and revealed that in patients treated with FOLFIRINOX, high expression of *SELENOP* and *G0S2* significantly correlated with good and poor overall survival, respectively. *SELENOP* encodes for the protein selenoprotein P (SeP), an essential protein for the transport of selenium into targeted tissues with antioxidant properties. Lower SeP expression has been found in clear cell renal cell carcinoma, breast, ovarian, colon and lung cancers [32]. Specifically in macrophages, reduced expression of SeP increased M2-polarized macrophages in a model of colitis-associated carcinogenesis [33]. Although to our knowledge, no studies indicate the expression of SeP in chemotherapy treated pancreatic cancer, in line with our findings of reduced SeP expression in macrophages under the influence of FOLFIRINOX treatment and CAFs, a study found a markedly diminished expression of SeP in the plasma of cervical cancer patients resistant to chemoradiotherapy [34]. Thus, the decreased expression of *SELENOP* could correlate with increased level of specific M2 macrophages and decreased sensitivity to FOLFIRINOX. On the other hand, *G0S2* encodes for the protein G0/G1 switch-2, initially thought to regulate cell cycle progression but has now been identified to also have major roles in apoptosis and lipid metabolism as a prominent inhibitor of adipose triglyceride lipase, blocking lipolysis [35, 36]. The overexpression of *G0S2* as a lipid-droplet associated protein was observed in pancreatic cancer samples compared to normal pancreas but was not significantly associated with overall survival of patients [37]. Here, we show that low *SELENOP* and high *G0S2* gene expression, found specifically in macrophages in coculture with CAFs treated with FOLFIRINOX, correlate with poor overall survival in FOLFIRINOX-treated patients. Additionally, the combined scores of expression of these two genes are significantly better predictors of poor prognosis than each gene alone, demonstrating a synergistic effect of this gene signature. Overall, we revealed a specific switch in the above genes’ expression in macrophages, highlighting a potentially specific M2 macrophage sub-type present in PDAC tumors under the influence of CAFs, in presence of FOLFIRINOX, and demonstrated that this switch in expression is associated with patient survival, in response to FOLFIRINOX. Further mechanistic and correlative studies would be required to better understand the influence of these genes in promoting M2 macrophage activity in the TME under chemotherapy.

## Conclusions

Altogether, the present study highlights macrophages reprogramming by CAFs under FOLFIRINOX to a stronger M2-macrophage phenotype, with a unique gene expression profile predictive of survival in FOLFIRINOX-treated patients. Importantly, our study potentially enriches the clinical toolbox with biomarkers able to predict the FOLFIRINOX-responsive PDAC patients. Those biomarkers would not be based on tumor cell activities but on the specific cellular composition of TME, and more specifically of M2 macrophage subtypes. Future studies could investigate signals secreted by untreated or FOLFIRINOX-treated CAFs to identify upstream factors involved in macrophage remodeling, then determine the impact of these modulated macrophages on tumor cells chemosensitivity, to explore the mechanistic actions of differentially expressed genes in these macrophages. Determining specific CAFs subtypes associated with such M2 macrophages reprogramming could also drastically improve our understanding of TME in FOLFIRINOX response. Evidently, and besides the biomarker field deepened in this study, enlightenment on the role of macrophages and CAFs crosstalk could be a source of potential novel therapeutic targets aimed to alleviate chemoresistance mechanisms in PDAC.

### Supplementary Information

**Additional file 1: Supplementary Fig. 1. **Representative FACs plots of expression of M1 markers CD80, CD83 and CD86 (A) or M2 markers CD200R, CD209 and CD206 (B) in, respectively M1 or M2, macrophages with or without CAFs. Representative FACs plots of expression of M1 markers CD80, CD83 and CD86 (C) or M2 markers CD200R, CD209 and CD206 (D) in, respectively M1 or M2, macrophages with or without CAFs coculture and with or without FOLFIRINOX treatment.

**Additional file 2:** **Supplementary Fig. 2. **Median fold change of expression of selected significantly differentially expressed cytokines and chemokines secreted by M2 macrophages, M2 macrophages in coculture with CAFs, M2 macrophages treated with FOLFIRINOX, and M2 macrophages in coculture with CAFs and treated with FOLFIRINOX.

**Additional file 3: Supplementary Fig. 3.** RNA sequencing analysis of M2 macrophages cocultured with PDAC CAFs and/or treated with FOLFIRINOX and the differential impact of PDAC CAFs on M2 macrophage gene expression profile under FOLFIRINOX treatment. Heat maps (left panel) and associated gene enrichment analyses (right panel) of differential gene expression between (A) M2 macrophages and M2 macrophages cocultured with CAFs (B) M2 macrophages treated with FOLFIRINOX vs. untreated (C) M2 macrophages treated with FOLFIRINOX in monoculture vs. in coculture with CAFs (D) M2 macrophages in cocultures with CAFs treated and untreated with FOLFIRINOX. Genes were considered significantly differentially regulated if *p*-value < 0.05 and log_2_fold-change > 1.

**Additional file 4:** **Supplementary Fig. 4.** (A) Enriched Ontology clusters in group “0”. (B) Distribution of mRNA expression levels (log_2_) of the top 10 genes across the 938 primary PDAC clinical samples.

**Additional file 5:** **Supplementary Table 1.** List of PDAC data sets included.

## Data Availability

Data are available in a public, open access repository. All data relevant to the study are included in the article, uploaded as online supplementary information or have been deposited in the public, open access repository NCBI’s Gene Expression Omnibus and are accessible through GEO Series accession number “GSE226448”.
