# Supplementary figures and images for: Macrophages reprogramming driven by cancer-associated fibroblasts under FOLFIRINOX treatment correlates with shorter survival in pancreatic cancer
Source: Cell Commun Signal. 2024 Jan 2;22:1. doi: 10.1186/s12964-023-01388-7 (PMC10759487; doi:10.1186/s12964-023-01388-7)

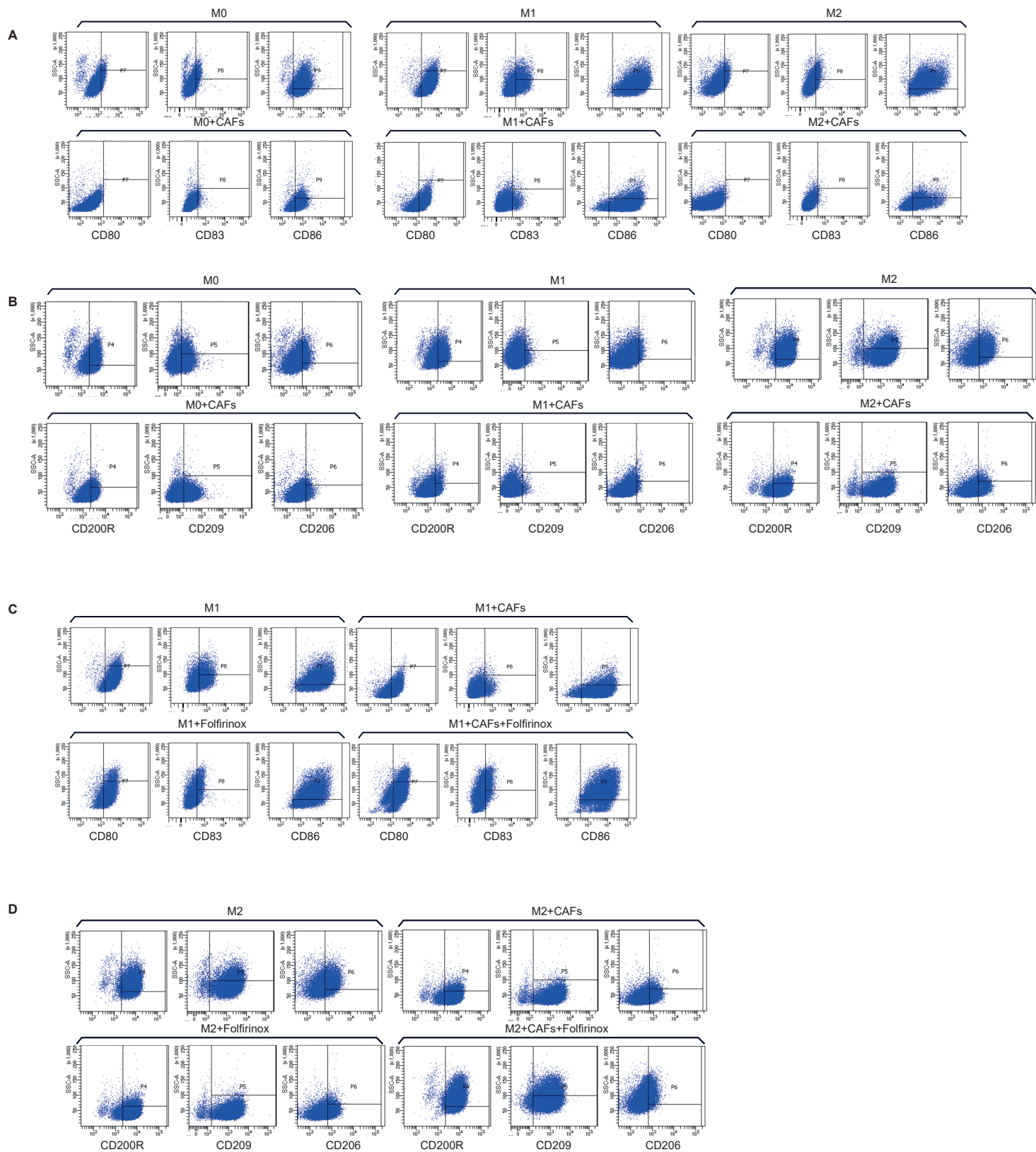

Supplement: Supplementary file 2 — Additional file 1: Supplementary Fig. 1. Representative FACs plots of expression of M1 markers CD80, CD83 and CD86 (A) or M2 markers CD200R, CD209 and CD206 (B) in, respectively M1 or M2, macrophages with or without CAFs. Representative FACs plots of expression of M1 markers CD80, CD83 and CD86 (C) or M2 markers CD200R, CD209 and CD206 (D) in, respectively M1 or M2, macrophages with or without CAFs coculture and with or without FOLFIRINOX treatment. [file 12964_2023_1388_MOESM1_ESM.pdf]

A

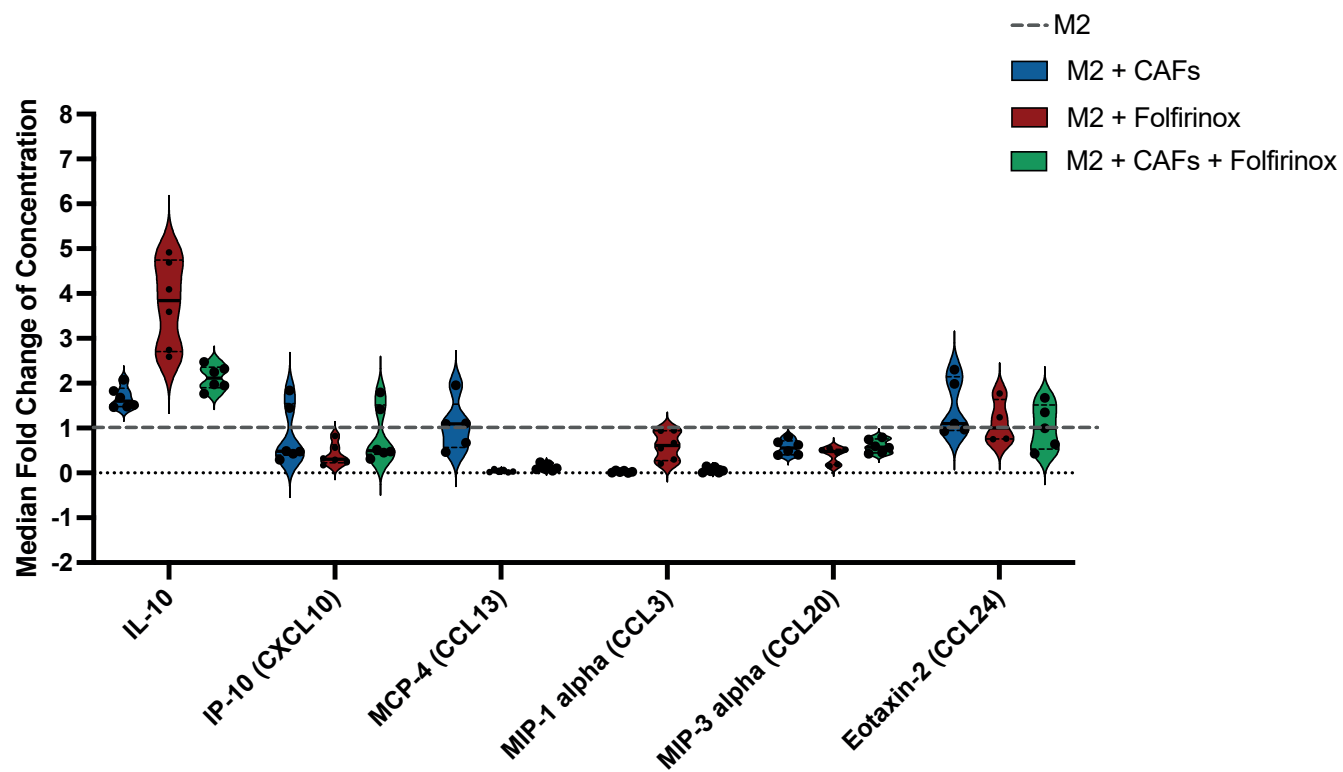

Supplement: Supplementary file 3 — Additional file 2: Supplementary Fig. 2. Median fold change of expression of selected significantly differentially expressed cytokines and chemokines secreted by M2 macrophages, M2 macrophages in coculture with CAFs, M2 macrophages treated with FOLFIRINOX, and M2 macrophages in coculture with CAFs and treated with FOLFIRINOX. [file 12964_2023_1388_MOESM2_ESM.pdf]

A

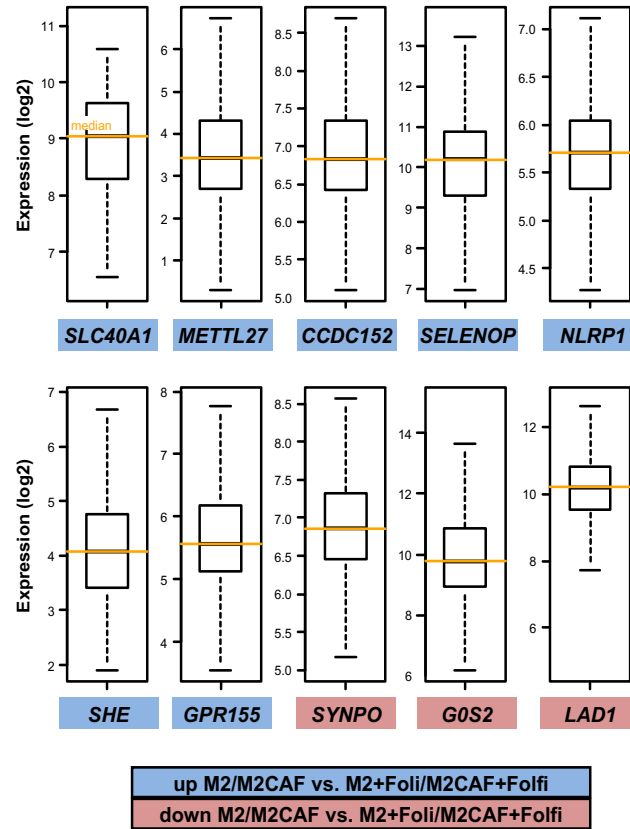

B

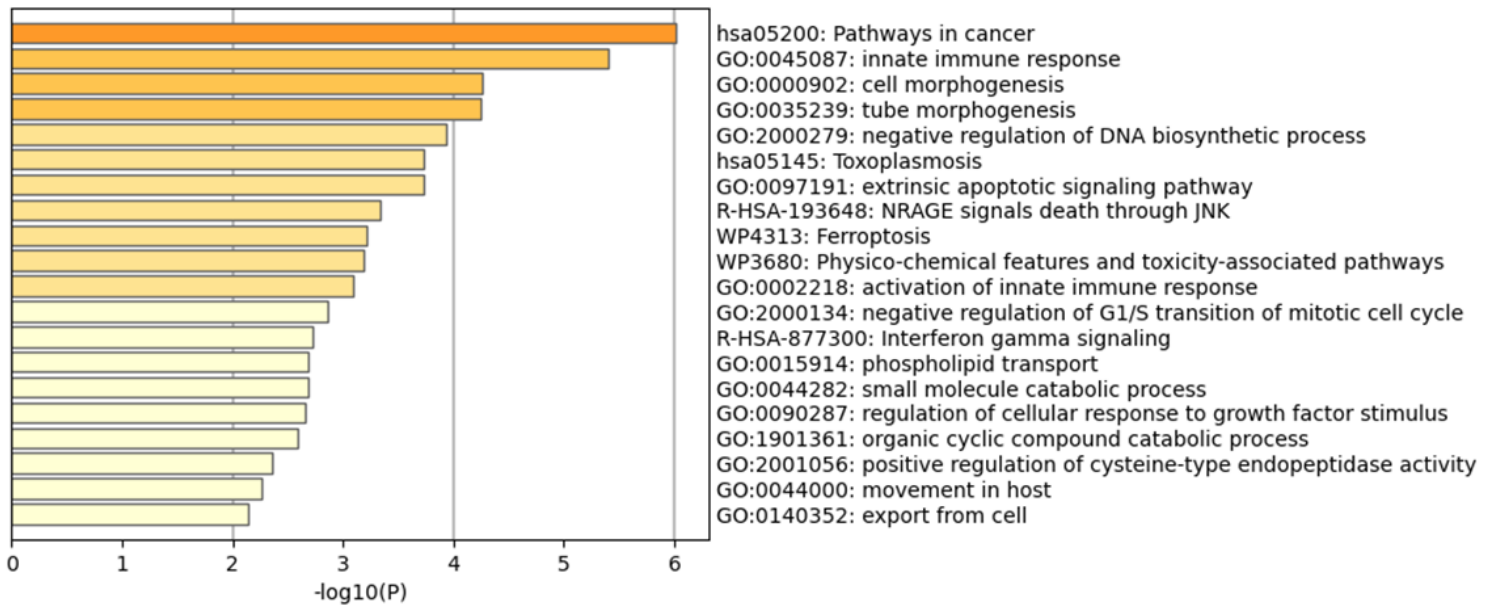

Supplement: Supplementary file 5 — Additional file 4: Supplementary Fig. 4. (A) Enriched Ontology clusters in group “0”. (B) Distribution of mRNA expression levels (log2) of the top 10 genes across the 938 primary PDAC clinical samples. [file 12964_2023_1388_MOESM4_ESM.pdf]
