# Supplementary material for: Macrophages reprogramming driven by cancer-associated fibroblasts under FOLFIRINOX treatment correlates with shorter survival in pancreatic cancer
Source: Cell Commun Signal. 2024 Jan 2;22:1. doi: 10.1186/s12964-023-01388-7 (PMC10759487; doi:10.1186/s12964-023-01388-7)
Supplement: Supplementary file 6 — Additional file 5: Supplementary Table 1. List of PDAC data sets included. [file 12964_2023_1388_MOESM5_ESM.pdf]

Supplemental Table 1: List of PDAC data sets included

Reference	Source of data	Technological platform	N° of probe sets/genes	Primary PDAC samples included in the present analysis
Badea et al., Hepatogastroenterology 2008	GEO database, GSE15471	Affymetrix, array U133 Plus 2.0	54K	36
van den Broeck et al., J Exp Clin Cancer Res 2012	GEO database, GSE42952	Affymetrix, array U133 Plus 2.0	54K	12
Zhang et al., PLoS One 2012 & Clin Cencer Res 2013	GEO database, GSE28735	Affymetrix, array Gene 1.0 ST	33K	45
Lunardi et al., Oncotarget 2014	GEO database, GSE55643	Agilent, array 4x44K G4112F (014850)	44K	45
Park et al., Mod Pathol 2014	GEO database, GSE43795	Illumina, array Human HT-12 V4.0	48K	6
Winter et al., PLoS Comput Biol 2012	Array-Express database, E-MEXP-2780	Affymetrix, array U133 Plus 2.0	54K	30
Grutzmann et al., Neoplasia 2004	Array-Express database, E-MEXP-950	Affymetrix, array U133 A+B	22K+22K	11
TCGA, PAAD	TCGA portal, https://tcga-data.nci.nih.gov	Illumina, RNA sequencing V2	25K	150
Straford et al., PLoS Med 2010	GEO database, GSE21501	Agilent, array 4x44K G4112F (014850)	44K	132
Monzon et al., Clin Oncol 2009	GEO database, GSE12630	Affymetrix, array U133 A	22K	24
Bailey et al., Nature 2016	European Genome-phenome Archive (EGA), EGAS00001000154	Illumina, RNA sequencing HiSeq	18K	96
Chen et al., PLoS ONE 2015	GEO database, GSE57495	Affymetrix, Rosetta/Merck RSTA Custom 2.0	60K	63
Collisson et al., Nat Med. 2011	GEO database, GSE17891	Affymetrix, array U133 Plus 2.0	54K	27
Chaika et al., PLOSOne 2012	GEO database, GSE34153	Agilent, array 4x44K G4112F (014850)	44K	15
ICGC, PACA CA (2019)	https://dcc.icgc.org/projects/PACA-CA	Illumina, RNA sequencing HiSeq	19K	195
Kirby et al., Mol Oncol. 2016	GEO database, GSE79670	Illumina, RNA sequencing HiSeq	49K	51
			**TOTAL**	**938**
