# Supplementary material for: Macrophages reprogramming driven by cancer-associated fibroblasts under FOLFIRINOX treatment correlates with shorter survival in pancreatic cancer
Source: Cell Commun Signal. 2024 Jan 2;22:1. doi: 10.1186/s12964-023-01388-7 (PMC10759487; doi:10.1186/s12964-023-01388-7)
Supplement: Supplementary file 4 — Additional file 3: Supplementary Fig. 3. RNA sequencing analysis of M2 macrophages cocultured with PDAC CAFs and/or treated with FOLFIRINOX and the differential impact of PDAC CAFs on M2 macrophage gene expression profile under FOLFIRINOX treatment. Heat maps (left panel) and associated gene enrichment analyses (right panel) of differential gene expression between (A) M2 macrophages and M2 macrophages cocultured with CAFs (B) M2 macrophages treated with FOLFIRINOX vs. untreated (C) M2 macrophages treated with FOLFIRINOX in monoculture vs. in coculture with CAFs (D) M2 macrophages in cocultures with CAFs treated and untreated with FOLFIRINOX. Genes were considered significantly differentially regulated if p-value < 0.05 and log2fold-change > 1. [file 12964_2023_1388_MOESM3_ESM.pdf]

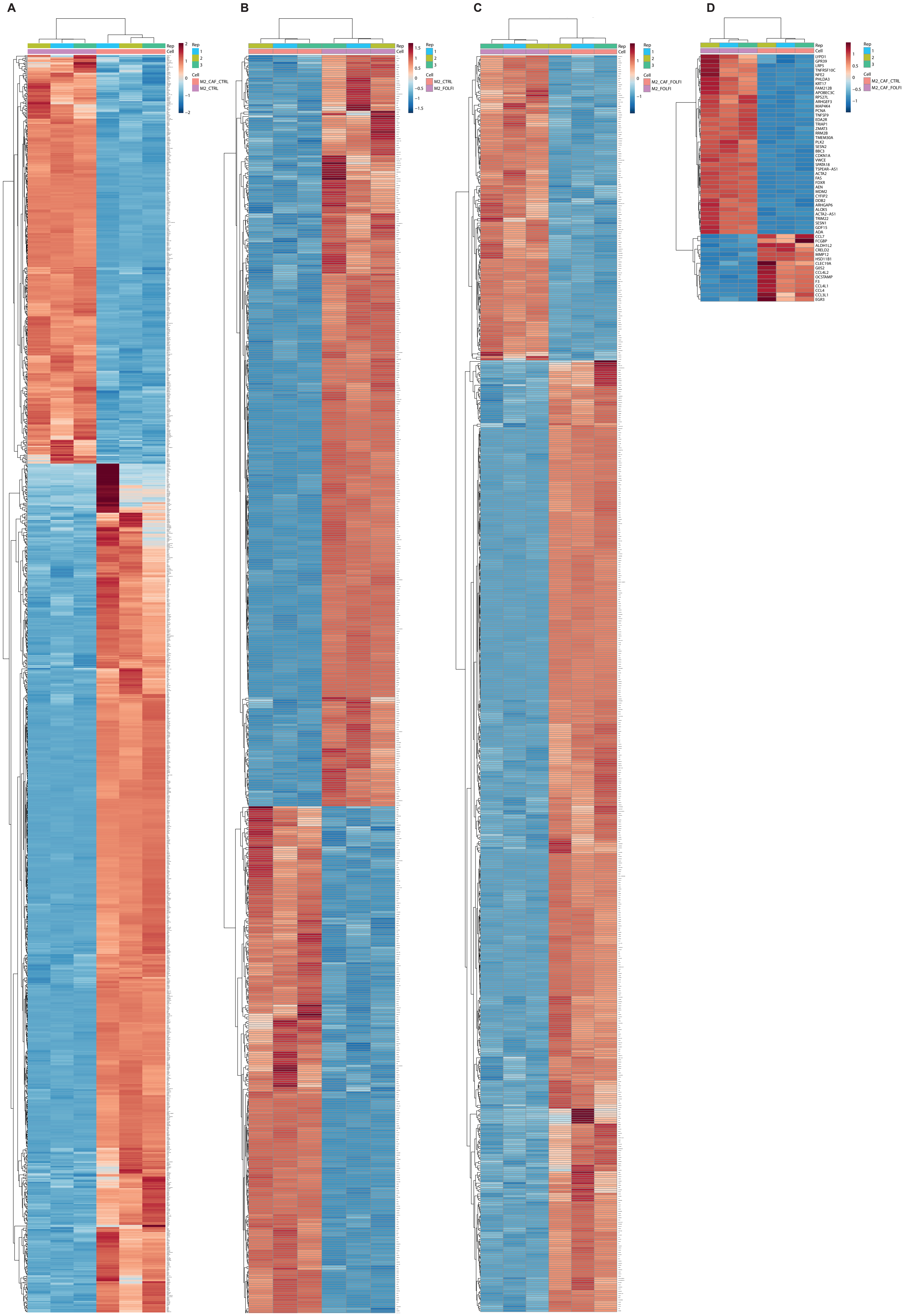
A
Rep
Cell
2
1
0
−1
−2
Rep
1
2
3
Cell
M2_CAF_CTRL
M2_CTRL
B
Rep
Cell
1.5
1
0.5
0
−0.5
−1
−1.5
Rep
1
2
3
Cell
M2_CTRL
M2_FOLFI
C
Rep
Cell
1
0
−1
Rep
1
2
3
Cell
M2_CAF_FOLFI
M2_FOLFI
D
Rep
Cell
LYPD1
GPR39
LRP5
TNFRSF10C
NFE2
PHLDA3
KRT17
FAM212B
APOBEC3C
RPS27L
ARHGEF3
MAP4K4
PCNA
TNFSF9
EDA2R
TRIAP1
ZMAT3
RRM2B
TMEM30A
PLK2
SESN2
BBC3
CDKN1A
VWCE
SPATA18
TSPEAR−AS1
ACTA2
FAS
FDXR
AEN
MDM2
CYFIP2
DDB2
ARHGAP6
ALOX5
ACTA2−AS1
TRIM22
SESN1
GDF15
ADA
CCL7
FCGBP
ALDH1L2
CRELD2
MMP12
HSD11B1
CLEC19A
G0S2
CCL4L2
OCSTAMP
F3
CCL4L1
CCL4
CCL3L1
EGR3
1
0.5
0
−0.5
−1
Rep
1
2
3
Cell
M2_CAF_CTRL
M2_CAF_FOLFI
